# Expanding the Repertoire of Modified Vaccinia Ankara-Based Vaccine Vectors via Genetic Complementation Strategies

**DOI:** 10.1371/journal.pone.0005445

**Published:** 2009-05-06

**Authors:** David A. Garber, Leigh A. O'Mara, Jun Zhao, Sailaja Gangadhara, InChul An, Mark B. Feinberg

**Affiliations:** 1 Emory Vaccine Center, Emory University, Atlanta, Georgia, United States of America; 2 Department of Medicine, Emory University, Atlanta, Georgia, United States of America; 3 Department of Microbiology and Immunology, Emory University, Atlanta, Georgia, United States of America; Institut Pasteur Korea, Republic of Korea

## Abstract

**Background:**

Modified Vaccinia virus Ankara (MVA) is a safe, highly attenuated orthopoxvirus that is being developed as a recombinant vaccine vector for immunization against a number of infectious diseases and cancers. However, the expression by MVA vectors of large numbers of poxvirus antigens, which display immunodominance over vectored antigens-of-interest for the priming of T cell responses, and the induction of vector-neutralizing antibodies, which curtail the efficacy of subsequent booster immunizations, remain as significant impediments to the overall utility of such vaccines. Thus, genetic approaches that enable the derivation of MVA vectors that are antigenically less complex may allow for rational improvement of MVA-based vaccines.

**Principal Findings:**

We have developed a genetic complementation system that enables the deletion of essential viral genes from the MVA genome, thereby allowing us to generate MVA vaccine vectors that are antigenically less complex. Using this system, we deleted the essential uracil-DNA-glycosylase (*udg*) gene from MVA and propagated this otherwise replication-defective variant on a complementing cell line that constitutively expresses the poxvirus *udg* gene and that was derived from a newly identified continuous cell line that is permissive for growth of wild type MVA. The resulting virus, MVAΔ*udg*, does not replicate its DNA genome or express late viral gene products during infection of non-complementing cells in culture. As proof-of-concept for immunological ‘focusing’, we demonstrate that immunization of mice with MVAΔ*udg* elicits CD8+ T cell responses that are directed against a restricted repertoire of vector antigens, as compared to immunization with parental MVA. Immunization of rhesus macaques with MVAΔ*udg-gag*, a *udg* **^−^** recombinant virus that expresses an HIV subtype-B consensus *gag* transgene, elicited significantly higher frequencies of Gag-specific CD8 and CD4 T cells following both primary (2–4-fold) and booster (2-fold) immunizations as compared to the *udg* ^+^ control virus MVA-*gag*, as determined by intracellular cytokine assay. In contrast, levels of HIV Gag-specific antibodies were elicited similarly in macaques following immunization with MVAΔ*udg-gag* and MVA-*gag*. Furthermore, both *udg* **^−^** and *udg* ^+^ MVA vectors induced comparatively similar titers of MVA-specific neutralizing antibody responses following immunization of mice (over a 4-log range: 10^4^–10^8^ PFU) and rhesus macaques. These results suggest that the generation of MVA-specific neutralizing antibody responses are largely driven by input MVA antigens, rather than those that are synthesized *de novo* during infection, and that the processes governing the generation of antiviral antibody responses are more readily saturated by viral antigen than are those that elicit CD8+ T cell responses.

**Significance:**

Our identification of a spontaneously-immortalized (but not transformed) chicken embryo fibroblast cell line (DF-1) that is fully permissive for MVA growth and that can be engineered to stably express MVA genes provides the basis for a genetic system for MVA. DF-1 cells (and derivatives thereof) constitute viable alternatives, for the manufacture of MVA-based vaccines, to primary CEFs – the conventional cell substrate for MVA vaccines that is not amenable to genetic complementation strategies due to these cells' finite lifespan in culture. The establishment of a genetic system for MVA, as illustrated here to allow *udg* deletion, enables the generation of novel replication-defective MVA mutants and expands the repertoire of genetic viral variants that can now be explored as improved vaccine vectors.

## Introduction

Modified Vaccinia virus Ankara (MVA), an attenuated strain of vaccinia virus that was originally developed as a smallpox vaccine, was obtained following extensive serial passage on primary chicken embryo fibroblasts (CEFs) [1]. During this process of attenuation, MVA underwent deletion of 31 kb (∼15%) of its genome, as compared to its parental strain, including a number of genes that contribute to viral evasion from host immune responses and that determine virus host range [2], [3]. As a result, MVA is unable to replicate productively in most mammalian cell types, including primary human cells. This block occurs at the relatively late stage of virion assembly and maturation (ie following expression of early (E), intermediate (I), and late (L) viral genes) [4], [5], [6], [7]. The resulting inability of MVA to undergo more than one infection cycle in a human host has imbued this virus with inherent safety that was demonstrated historically through the immunization of ∼120,000 individuals during the smallpox eradication campaign. More recently, the safety of MVA has been demonstrated in pre-clinical studies of immune-deficient mice and immune-suppressed macaques [8], [9] and in phase-I clinical trial evaluations of MVA as a next-generation smallpox vaccine [10].

The desirable safety profile exhibited by MVA, in concert with its ability to express high levels (and large numbers) of foreign genes, has rendered MVA a leading candidate for evaluation as a vaccine vector against an array of infectious diseases and human cancers. On a number of different fronts, MVA-based vaccines against HIV/AIDS [11], [12], [13], [14], [15], [16], malaria [17], [18], tuberculosis [19], [20], HPV-induced CIN [21], [22], and melanoma [23] are being evaluated in human clinical trials. Such broad interest to develop a diverse array of MVA-based vaccines provides substantial opportunities to engineer MVA vectors to enhance their immunogenicity – but, to date, these have been largely unrealized.

The utility of MVA-based vaccines to prime immune responses against heterologous antigens appears to be limited due to unfavorable competition for immunodominance between the relatively large number of vector-specific gene products (177 [3]) and the dramatically smaller number of intended vaccine antigens [24]. Moreover, repeated administration of recombinant MVA vaccine vectors typically results in an increasingly diminished efficacy of such booster immunizations, presumably due to the elicitation of vector-specific neutralizing antibody responses [25], [26], [27]. Disappointing results from a phase I clinical trial of an MVA-based AIDS vaccine [28], [29] suggest that there is a substantial need to better understand the mechanisms governing antigen presentation [30], [31], immunodominance [32], and the generation of vector-specific humoral immunity [33] in order to improve upon the immunogenicity of currently available MVA vaccine vectors.

One approach towards overcoming these limitations is to block progression of the viral replication cycle at an earlier stage than normally occurs with MVA infection. This genetic restriction of viral gene expression would effectively reduce the overall number of irrelevant, but potentially immunodominant, MVA gene products that are synthesized during infection. These include virion structural proteins, which are synthesized with predominantly late kinetics during infection, that also contain epitopes targeted by neutralizing antibody responses. In this way, deletion of an essential MVA gene(s) could augment the immunogenicity of MVA-based vaccine vectors by effectively reducing the antigenic complexity of the vector, which could focus cellular and/or humoral immune responses away from irrelevant (or even undesirable) antigenic targets of the vector itself and toward the antigen of interest.

Replication-defective mutants of other large DNA viruses, including herpes simplex virus [34], [35], [36], [37], adenovirus [38], and vaccinia virus [39], have been produced for use as vaccine vectors, but this approach has not yet been feasible with MVA. This absence of a system for genetic complementation of deletion (or mutation) of essential genes from MVA derives in large part from the fact that MVA is routinely propagated on primary CEFs, which, due to their finite lifespan in culture, are not suitable for long-term (multi-passage) genetic complementation.

In the present study, we describe the establishment of a genetic system to complement deletion of essential MVA genes and its use to create a recombinant MVA from which the uracil-DNA-glycosylase gene has been deleted. This virus, MVAΔ*udg*, exhibits an immune-response phenotype that is distinct from wild type MVA. This system was predicated on the identification of an immortalized CEF-derived cell line (UMNSAH/DF-1, “DF-1” [40], [41]) that supports high-level growth of MVA and that could be engineered to constitutively express *udg*, and presumably, other MVA genes. Importantly, DF-1 cells (and derivatives thereof) are attractive alternatives to primary CEFs for large-scale production of MVA-based vaccines because of their origin through spontaneous immortalization, rather than oncogenic transformation, of embryonic fibroblasts derived from chickens that were free from endogenous retroviruses [40], [41]. As a proof-of-concept, we demonstrate that immunization of mice with MVAΔ*udg* elicits CD8+ T cell responses against fewer MVA vector antigens than does wild type MVA, thereby constituting an effective focusing of the antiviral CD8+ T cell repertoire toward antigens that are expressed early, rather than late, during the MVA replication cycle. We further show that immunization of rhesus macaques with MVAΔ*udg-gag*, a *udg* **^−^** vector that expresses an HIV *gag* transgene from an early viral promoter, elicits significantly higher (2–4-fold) frequencies of HIV Gag-specific CD8 and CD4 T cells following primary and booster immunizations, as compared to a *udg* ^+^ control (MVA-*gag*). In contrast to its positive effects on transgene-specific T cell responses, deletion of *udg* from recombinant MVA vectors had no discernable impact on the magnitudes of transgene-specific antibody responses that were elicited in rhesus macaques or on the MVA-specific antibody responses that were elicited in either mice or macaques.

## Results

### The DF-1 fibroblast cell line supports high-level growth of MVA

**Figure 1:**
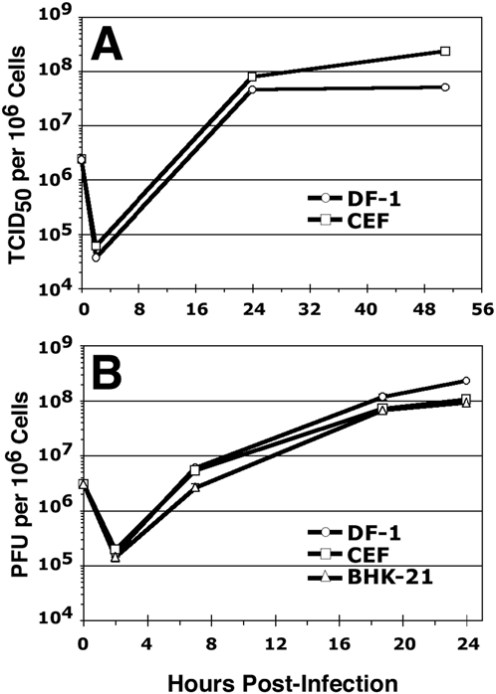
DF-1 cells support high-level growth of MVA. (A) MVA yields following infection of DF-1 fibroblasts and 1° CEFs at MOI = 3. Data represent the means of duplicate samples. (The MVA stock was grown and titered on 1° CEFs). (B) MVA yields following infection of DF-1 fibroblasts, 1° CEFs, and BHK-21 cells at MOI = 3. Data represent the means of triplicate samples ±1 standard deviation. (The MVA stock was grown and titered on DF-1 cells).

Given that an immortalized cell line is a prerequisite for any viral genetic complementation strategy, we first sought to identify a cell line that was able to support MVA replication efficiently and that might also be suitable for ultimate manufacture of licensable MVA-based vaccines. Based on their derivation and character, we reasoned that the CEF-derived DF-1 cell line might be a promising cell substrate for MVA vector preparation and propagation. We first assayed the ability of DF-1 fibroblasts to support the growth of MVA in comparison with two other permissive cell types, primary CEFs and BHK-21 cells (Figure 1). Cells were infected with MVA at a ratio of 3 infectious units per cell, and MVA yields were calculated via TCID_50_ determination on primary CEFs (Figure 1A) or via plaque assay on DF-1 cells (Figure 1B) at the indicated times following infection. Following the expected period of viral eclipse at 2 hours post-infection, MVA infection resulted in the net production of approximately 10^8^ infectious units per million DF-1 cells by 24 hours post-infection. The yields of MVA from infected DF-1 cells were equivalent to those obtained following infection of primary CEF or BHK-21 cells (Figure 1A, B). Thus, DF-1 fibroblasts are fully permissive for MVA growth and support virus growth to levels that are comparable to those obtained through propagation of MVA on primary CEFs.

### Zeocin-resistance/GFP-positivity allows for rapid isolation of MVA recombinants

A number of methods that facilitate selection or screening for recombinant vaccinia virus are also applicable for the isolation of recombinant MVA. These include expression of drug resistance markers (*gpt* [42], [43], *neo* [44], [45], *puro* [46]) whose transient or stable expression from the viral genome allow for enrichment of drug-resistant recombinant viruses, and expression of genes (*lacZ* , *gfp* [45], *gus* [47]) whose products may be detected via fluorescence or colorimetric assays to expedite the identification and isolation of recombinant viruses. We sought to evaluate the use of zeocin [48], an antibiotic whose mechanism of action rests upon its ability to intercalate into DNA and subsequently cleave DNA molecules, as a new antibiotic selection to facilitate the prompt isolation of MVA and vaccinia recombinants. Zeocin was found to be a potent inhibitor of MVA growth. Addition of this antibiotic to the culture medium of MVA-infected DF-1 cells at concentrations ranging from 50–200 µg/ml reduced MVA yields between 82–98% as compared to growth in the absence of the drug (not shown).

**Figure 2:**
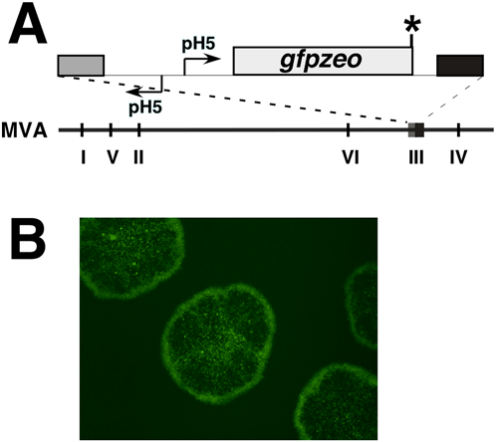
Construction and characterization of MVA-GZ. (A) Genome map of MVA recombinant MVA-*gz* (top), which encodes *gfpzeo* (*gz*) under the control of an early modified H5 promoter (pH 5). Roman numerals (I-VI) represent the sites of the major genomic deletions in MVA as compared to its parental strain [3]. * = vaccinia virus early transcriptional stop signal (5′-TTTTTCT-3′) [70]. (B) Recombinant (MVA-*gz*) plaques visualized via fluorescence microscopy as GFP^+^ plaques on DF-1 cells at 4 days following infection; original magnification = 4X.

To demonstrate that zeocin selection could be used to facilitate isolation of recombinant viruses, we recombined a DNA cassette that directs early expression of the *gfpzeo* fusion gene, which encodes an N-terminal green fluorescent protein (GFP) in frame with a C-terminal zeocin-resistance polypeptide (*Sh ble* gene product [49] ), at deletion site III of the MVA genome (Figure 2A) and selected for zeocin-resistant recombinants. Microscopic visualization of recombinant GFP^+^ plaques at 2 days following infection of DF-1 cells in the presence of zeocin (Figure 2B) enabled rapid plaque isolation and subsequent iterative plaque purification of the viral recombinant MVA-*gz* (Figure 2A). Thus, simultaneous drug selection and visual screening for GFPZEO**^+^** plaques expedites the isolation and preparation of recombinant MVA viruses.

### Generation and characterization of MVAΔ*udg*

**Figure 3:**
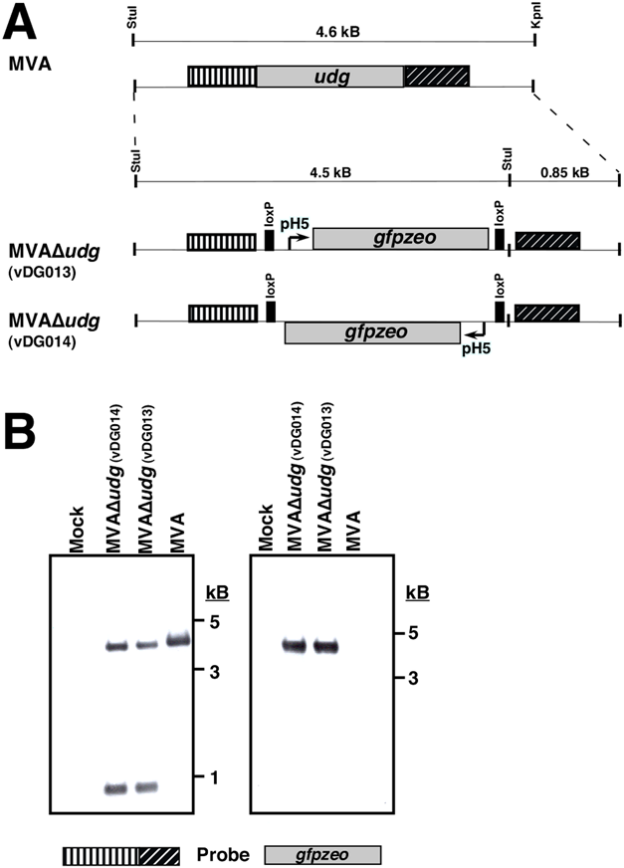
Isolation of MVAΔ*udg* recombinant viruses. (A) Genome maps of wild type MVA and *udg*-deletion MVA recombinants with restriction fragment lengths in kilobases (kB). The StuI and KpnI restriction sites in wild type MVA denote genomic nucleotide positions 89,347 and 93921, respectively. (B) Diagnostic Southern blots that confirm genotypes of MVAΔ*udg* isolates.

Vaccinia virus-encoded uracil-DNA-glycosylase is essential for VV growth. [50], [51], [52], [53], [54]. Because MVA is derived from vaccinia virus (strain CVA) and UDG^MVA^ shares complete amino acid identity with UDG encoded by other vaccinia strains (Copenhagen, Western Reserve), it was expected that *udg* would also be essential for MVA growth and that its deletion would require a genetic complementation strategy. An initial step towards deleting *udg* from MVA was to generate an MVA-permissive cell line that also expresses UDG at levels sufficient to complement genomic deletion of *udg*. MVA-permissive DF-1 cells (Fig. 1) were transfected with a *udg*-expression plasmid pCAN*udg* (see materials and methods) and G418-resistant cell clones were screened for their ability to complement the growth of *ts*4149, a vaccinia virus mutant that harbors a temperature sensitive mutation in the *udg* (D4R) gene, at the non-permissive temperature of 39.5°C (not shown). A clonal cell line that supported high-level replication of *ts*4149 at the non-permissive temperature was designated CAN20 and used as the cellular substrate to generate MVAΔ*udg* recombinants. To delete *udg* from MVA, plasmid transfer vectors pΔ*udg*dloxPH5*gz*-A, -B, which differ from each other only in the orientation of their pH5-*gfpzeo* expression cassettes, were constructed to direct replacement of the *udg* ORF with a loxP-flanked *gfpzeo* expression cassette. MVA-infected CAN20 cells were transfected with pΔ*udg*dloxPH5*gz*-A or pΔ*udg*dloxPH5*gz*-B (as described, materials and methods) and GFP^+^/zeocin-resistant recombinants were plaque purified through 4 rounds of zeocin selection. The genotypes of two independent MVAΔ*udg* recombinants, vDG013 and vDG014 (Figure 3A), that harbor *gfpzeo* expression cassettes in opposite orientations at the *udg* locus were confirmed via Southern blot and shown to be free from contamination with wild type (parental) MVA (Figure 3B).

### MVAΔ*udg* recombinants grow only on *udg*-complementing cells

**Figure 4:**
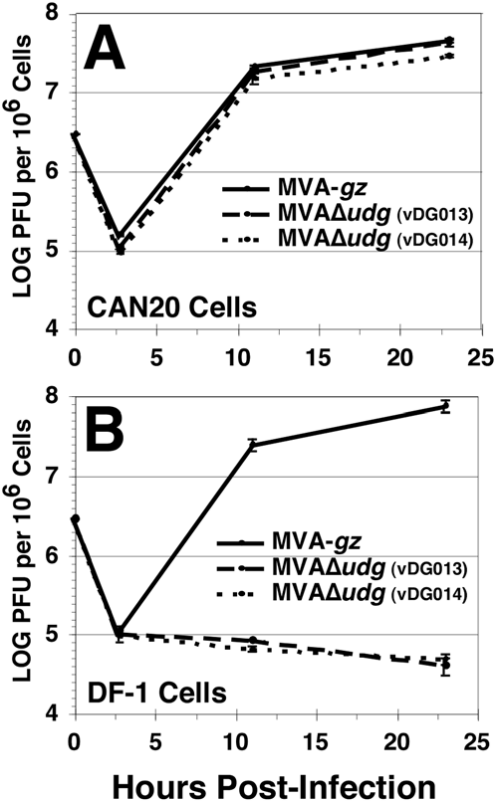
MVAΔ*udg* recombinants grow on the DF-1-derived *udg*-complementing cell line (CAN20), but do not grow on parental DF-1 cells. Yields of MVAΔ*udg* recombinants vDG013, vDG014, and *udg* ^+^ recombinant MVA-*gz* were determined at the indicated times following infection of CAN20 cells (A) or DF-1 cells (B) at a ratio of 3 PFU per cell. Cell cultures were frozen at indicated times following infection and subsequently thawed, sonicated, and clarified by centrifugation (800 *g*). Virus titers were determined via plaque assay on CAN20 cells. Data represent the means of duplicate samples; error bars represent the ranges.

To confirm that we had deleted an essential viral gene from MVA, we first compared the abilities of 2 independent MVAΔ*udg* isolates and MVA-*gz* (*udg* ^+^) to grow on UDG-complementing cells or parental DF-1 cells (Figure 4). MVA-*gz* is a *udg* ^+^ MVA recombinant that expresses the *gfpzeo* fusion gene (Figure 2B). UDG-complementing cells (CAN20 cells) (Figure 4A) or DF-1 fibroblasts (Figure 4B) were infected with MVAΔ*udg* isolates vDG013, vDG014, or with (*udg* **^+^**) MVA-*gz* at a virus/cell ratio of 3, and virus yields were determined by plaque assay on CAN20 cells at the indicated times following infection. Both MVAΔ*udg* isolates exhibited virus yields equivalent to those seen with (*udg* ^+^) MVA-*gz* during infection of CAN20 cells (Figure 4A). In contrast, neither MVAΔ*udg* isolate exhibited any net increase in virus yield during infection of non-complementing cells, whereas MVA-*gz* replicated to high titers of approximately 10^8^ PFU per million DF-1 cells, confirming that *udg* is essential for MVA growth.

### MVAΔ*udg* does not exhibit DNA replication or expression of viral late genes

**Figure 5:**
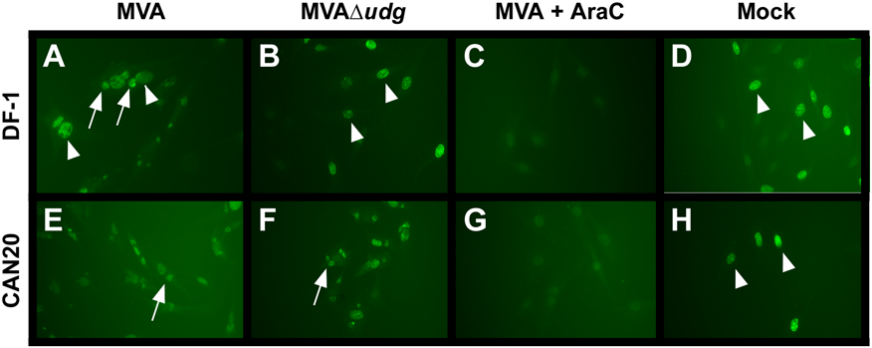
MVAΔ*udg* does not exhibit DNA replication during infection of non-complementing cells. DF-1 cells (A, B, C, D) and CAN20 cells (E, F, G, H) were infected either with MVA in the absence (A, E) or presence (C, G) of the DNA synthesis inhibitor AraC (150 µM), MVAΔ*udg* (vDG020) in the absence of AraC (B, F), or were mock infected (D, H) and labeled with BrdU between 2–6 hours following infection. Arrows denote cytoplasmic foci of viral DNA replication and arrowheads denote cell nuclei.

In vaccinia virus infections, UDG has an essential role in viral DNA synthesis that is independent of its glycosylase activity [54]. To confirm that deletion of *udg* from the MVA genome also results in a block to viral DNA replication, we monitored DNA replication via microscopic detection of bromodeoxyuridine (BrdU) incorporation into newly-synthesized DNA within infected cells. MVAΔ*udg* recombinant virus vDG020- , MVA- , or mock-infected UDG-complementing (CAN20) and non-complementing (DF-1) cells were labeled with BrdU between 2–6 hours following infection (Figure 5). Cytoplasmic foci containing newly synthesized MVAΔ*udg* DNA were observed only during infection of *udg*-complementing CAN20 cells (Figure 5F) and were not observed during MVAΔ*udg* infection of non-complementing DF-1 cells (Figure 5B). In contrast, MVA infection of both CAN20 and DF-1 cell lines resulted in the generation of cytoplasmic viral DNA replication centers (Figure 5A, 5E) that were absent when these infections were performed in the presence of araC, an inhibitor of viral DNA synthesis (Figure 5C, 5G).

**Figure 6:**
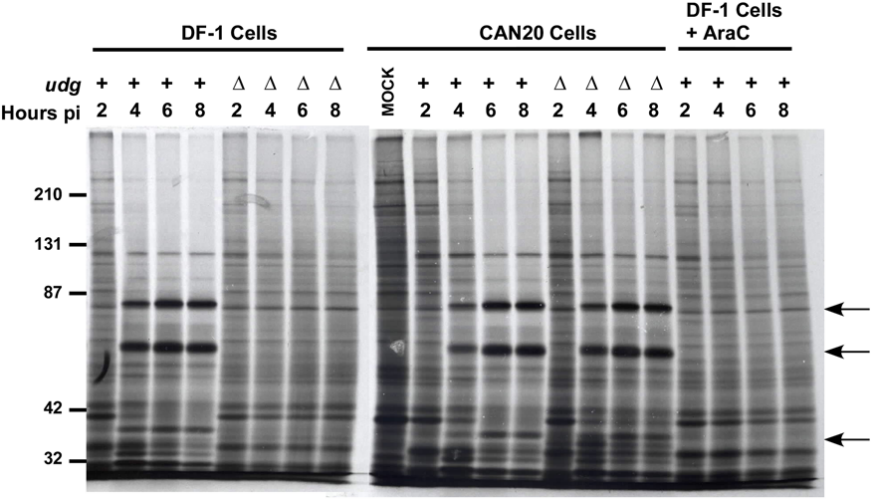
MVAΔ*udg* does not express viral late genes during infection of non-complementing cells in culture. DF-1 and *udg*-complementing (CAN20) cells were infected with MVA (*udg* ^+^) or MVAΔ*udg* isolate vDG014 (Δ) at MOI = 10 in the absence or presence of the DNA synthesis inhibitor AraC (150 µM), as indicated. Infected cell proteins were metabolically labeled with ^35^S-methionine for 30 min immediately prior to harvesting at indicated times post infection. Proteins were separated via SDS-PAGE and visualized by autoradiography. Arrows denote viral late gene products as defined via AraC-mediated inhibition of expression.

Because the expression of viral late genes is dependent upon viral DNA synthesis, we also determined the level of late gene expression during MVAΔ*udg* infection of either complementing or non-complementing cells. SDS-PAGE resolution of infected cell proteins that were radiolabeled with ^35^S-methionine during MVAΔ*udg* (vDG014)-, MVA(*udg* ^+^)-, or mock-infection of CAN20 and DF-1 cells demonstrated a defect in the production of the protein products of several late genes during MVAΔ*udg*-infection of non-complementing DF-1 cells (Figure 6).

### MVAΔ*udg* immunization elicits CD8+ T cell responses against fewer vector antigens than does parental MVA

**Figure 7:**
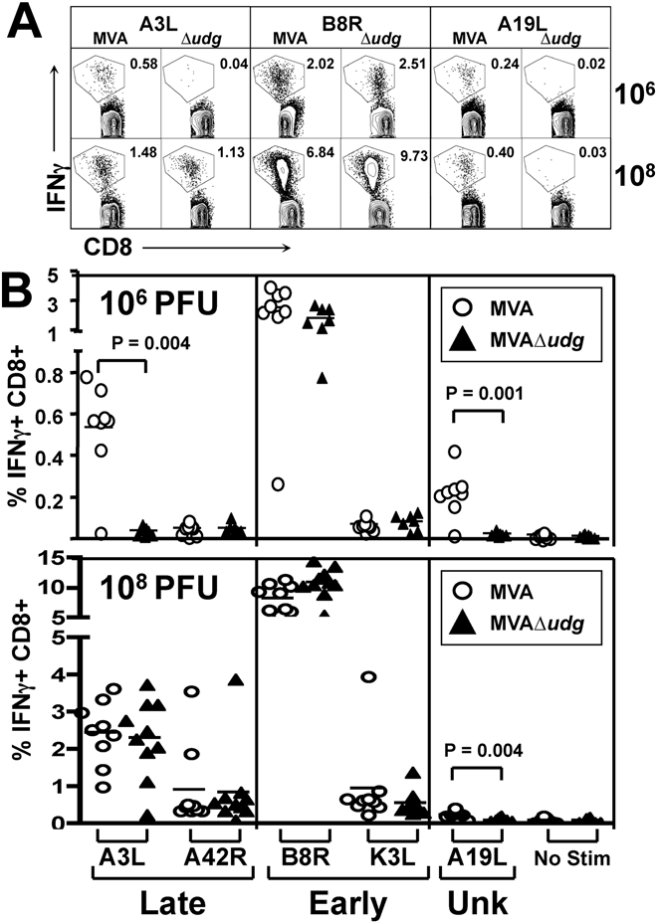
Immunization of mice with MVAΔ*udg* elicits CD8^+^ T cell responses directed against early, but not late viral gene products. (A) Representative intracellular cytokine staining (ICS) of peptide-stimulated splenocytes at 7 days following immunization with MVA or MVAΔ*udg*, as indicated. Splenocytes were stimulated *ex vivo* with 0.5 uM of A3L_270–277_, B8R_20–27_, or A19L_47–55_ peptide for 5 hours in the presence of GolgiPlug secretion inhibitor and stained with fluorescently-labeled antibodies for flow cytometric analysis. Plots represent data from individual mice and denote the percentages of IFNγ-positive CD3+CD8+ splenocytes expressed as fractions of their corresponding overall CD3+CD8+ splenocyte populations. (B) Splenocytes from mice immunized with MVA (circles) or MVAΔ*udg* (triangles) were analyzed by ICS assay (as above) following *ex vivo* stimulation with 0.5 µM A3L_270–277_, A42R_88–96_, B8R_20–27_, K3L_6–15_, or A19L_47–55_ peptide, or no stimulation, to determine the frequencies of antiviral CD8+ T cells present at 7 days following immunization. Data are organized by the kinetic class (Late, Early, or Unknown [Unk]) to which each viral gene belongs. Symbols represent data from individual mice; horizontal lines represent group means. Each dosage group (10^6^, 10^8^ PFU) presents data obtained from two independent immunization experiments. Statistical comparison of MVA vs MVAΔ*udg* groups, for each CD8+ T cell epitope, was performed via nonparametric Mann-Whitney analysis; only P-values ≤0.05 are shown.

Because MVAΔ*udg* was blocked at the E→L transition during infection of non-complementing cells in culture, we determined whether this abrogated *in vivo* CD8+ T cell responses directed against viral late gene products following immunization of mice. To measure these responses, mice were immunized with MVAΔ*udg* or parental MVA and their splenic CD8+ T cell responses characterized at 7 days post-immunization via intracellular cytokine staining (ICS) assay, which utilized an epitope panel comprised of recently-defined CD8+ determinants [55] representing both Early and Late viral antigens (Figure 7). Representative flow cytometry plots of CD8+ splenocytes that produce IFNγ following *ex vivo* peptide stimulation are shown (Figure 7A). The frequencies of epitope-specific CD8+ T cell responses, determined for individual mice, are shown following immunization with 10^6^ PFU (Figure 7B) or 10^8^ PFU (Figure 7C) of either MVAΔ*udg* or wild type MVA control, as indicated. Following immunization of mice at the lower dose (10^6^ PFU), MVAΔ*udg* elicited 14-fold and 9-fold lower frequencies of CD8+ T cells against the A3L_270–277_ (Late) and A19L_47–55_ (Unknown) determinants, respectively, than did MVA, which were determined to be statistically significant differences (Mann-Whitney test). In contrast, the average frequencies of CD8+ T cells directed against A42R_88–96_ (0.05% MVA, 0.05% MVAΔ*udg*), B8R_20–27_ (2.6% MVA, 1.9% MVAΔ*udg*), or K3L_6–15_ (0.07% MVA, 0.08% MVAΔ*udg*) were not significantly different between groups. Similarly, following immunization of mice at the higher dose (10^8^ PFU), both MVAΔ*udg* and MVA elicited similar frequencies of A42R_88–96_-, B8R_20–27_-, or K3L_6–15_-specific CD8+ T cells, which were ≥3-fold higher than the corresponding frequencies that were elicited following 10^6^ PFU immunization. Interestingly, immunization with MVAΔ*udg* at the high dose (10^8^ PFU) overcame the deficit of A3L_270–277_-restricted T cells that was observed following immunization with 10^6^ PFU MVAΔ*udg*, but did not abrogate the elicitation of a relatively lower frequency of A19L_47–55_-restricted CD8+ T cells, as compared to MVA (MVAΔ*udg* = 0.09%, MVA = 0.22%, average No Stim/limit of detection = 0.07%). Because the A3L protein is a structural component of the MVA virion (and therefore present in the viral inoculum), our observation that A3L_270–277_-restricted CD8+ T cells are elicited by MVAΔ*udg* only following a relatively high-dose immunization is most readily explained as a result of cross presentation of A3L antigen from the input virus, rather than *de novo* synthesized A3L antigen.

### Immunization of mice with MVAΔ*udg* or parental MVA elicits similar titers of vector-specific neutralizing antibodies

**Figure 8:**
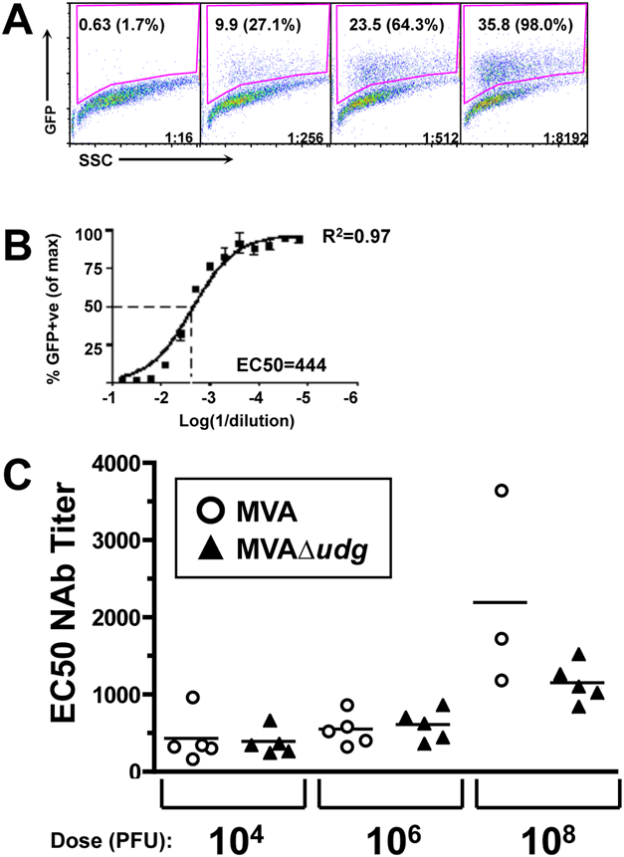
Immunization of mice with MVAΔ*udg* elicits MVA-specific neutralizing antibody responses that are of magnitudes similar to those elicited by MVA. (A) Representative flow cytometric data from the GFP fluorescence-based MVA neutralization assay. Serial dilutions of serum from a mouse immunized 28 days earlier with 10^6^ PFU MVA were mixed with a constant amount of GFP-expressing virus MVA-*gz* and incubated for 1 hour at 37°C. HeLa cells were then added to individual serum∶virus mixtures, incubated overnight at 37°C, and analyzed for GFP expression by flow cytometry. The gated percentages of GFP+ cells (shown) were also normalized to the average maximum response observed for cells infected with MVA-*gz* in the absence of test serum (normalized values expressed as percentages of the maximum response are shown parenthetically). Illustrative data representing serum dilutions 1∶16, 1∶256, 1∶512, and 1∶8,192, which constitute a subset of all serum dilutions analyzed, are shown. (B) Representative nonlinear regression analysis for the determination of EC50 neutralizing antibody (NAb) titers. Replicate normalized GFP+ response data from an individual mouse (described in (A)) was analyzed by non-linear regression. Goodness-of-fit value (R^2^) and the EC50 NAb titer, expressed as the dilution factor, are shown. (C) Mice were immunized once with the indicated doses of MVA (circles) or MVAΔ*udg* (triangles). Titers of MVA-specific neutralizing antibodies were determined, as described above, for serum samples collected 28 days following immunization. Symbols represent NAb titers that were determined for individual mice; horizontal lines represent group means. Statistical comparison of MVA vs MVAΔ*udg* groups, within each dosage group, was performed via nonparametric Mann-Whitney analysis and did not result in any significant (P≤0.05) differences.

Because many late viral genes encode virion structural components that embody B cell epitopes that engender neutralizing antibody (NAb) responses, we sought to determine whether abrogation of *de novo* late gene expression during *in vivo* infection, via *udg* deletion, might attenuate the overall magnitude of the resulting neutralizing antibody response that is generated against the MVA vector. Groups of mice were immunized with MVAΔ*udg* or MVA and their anti-MVA serum neutralizing antibody titers were determined 28 days later (Figure 8). Representative flow cytometry plots (Figure 8A) and corresponding serum dilution∶response curve (Figure 8B), which illustrate the titration of neutralizing antibodies in this assay, are shown and include goodness-of-fit (R^2^) and EC50 NAb titer values derived from the non-linear regression analysis. The MVA-specific NAb (EC50) titers, determined for individual mice, are shown following immunization with 10^4^, 10^6^, or 10^8^ PFU of either MVAΔ*udg* or MVA (Figure 8C). No statistical differences in neutralizing antibody titers existed between MVA and MVAΔ*udg* within any given immunization dosage group, or for either MVA or MVAΔ*udg* across immunization dosage groups. These data suggest that any contribution of *de novo* synthesized viral late gene products to the development of global vector-specific neutralizing antibody titers is negligible, and that such NAb responses are fully primed *in vivo* by relatively low amounts (ie containing ≤10^4^ PFU) of input viral inocula.

### Immunization of rhesus macaques with MVAΔ*udg-gag* elicits enhanced frequencies of CD8 and CD4 T cells that are specific for the expressed HIV *gag* transgene

**Figure 9:**
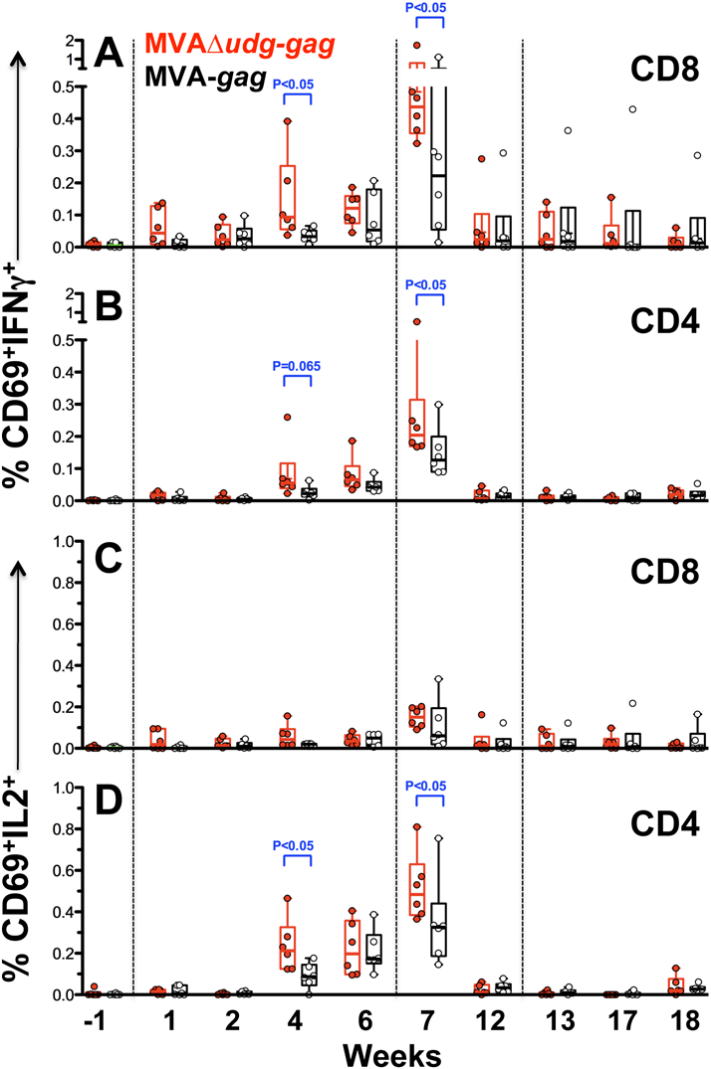
Immunization of rhesus macaques with MVAΔ*udg-gag* elicits significantly higher frequencies of HIV Gag-specific CD8 and CD4 T cells. Rhesus macaques (N = 6/group) were immunized at 0, 6, and 12 weeks with MVAΔ*udg-gag* or MVA-*gag* (2×10^8^ PFU per immunization). At the indicated times, PBMC samples were either stimulated *ex vivo* with a pool of matched overlapping HIV Gag peptides, or were not stimulated, and the frequencies of IFNγ- and IL2-producing CD8 and CD4 T cells were determined by intracellular cytokine staining/flow cytometric analysis as described. The frequencies of CD8 (A, C) and CD4 (B, D) T cells that co-expressed IFNγ (A, B) or IL2 (C, D) and the activation marker CD69 are shown. Symbols represent the means of replicate samples assayed for individual macaques; horizontal lines denote group medians. Statistical comparison of groups immunized with MVAΔ*udg-gag* vs MVA-*gag* was performed at each timepoint via non-parametric Mann-Whitney analysis. P-values <0.07 are indicated as shown. Immunizations are denoted by vertical dashed lines.

We next sought to determine whether an MVA vector that is deleted for *udg* elicits enhanced T cell responses against an expressed heterologous antigen, as compared to a *udg* ^+^ control virus, in a population of MHC-diverse rhesus macaques. Towards this end, we constructed MVAΔ*udg-gag* and MVA-*gag*, which commonly express a synthetic, human codon-optimized, HIV subtype-B consensus *gag* gene from an early viral promoter at MVA deletion site-III and differ only in their *udg* genotype, and used these viruses to immunize two groups of rhesus macaques (N = 6/group) with 2×10^8^ PFU of virus at 0, 6, and 12 weeks. At various times, both preceding and following immunization, we determined the frequencies of Gag-specific CD8 and CD4 T cells in PBMC via intracellular cytokine assay for the production of IFNγ and IL2 in response to *ex vivo* stimulation with a pool of matched overlapping Gag peptides (Figure 9). By 4 weeks following primary immunization, macaques immunized with MVAΔ*udg-gag* exhibited significantly greater frequencies of Gag-specific CD8 T cells, which produced IFNγ in response to Gag peptide stimulation, than did animals immunized with MVA-*gag* (MVAΔ*udg-gag*: range = 0.04–0.39, median = 0.094; MVA-*gag*: range = 0.01–0.07, median = 0.033) (Figure 9A). Relatively low frequencies of Gag-specific/IL2-producing CD8 T cells were observed following primary immunization with either virus (Figure 9B). Similarly, with regard to Gag-specific CD4 T cell responses, MVAΔ*udg-gag* elicited significantly higher frequencies of IL2-producing CD4 T cells at 4 weeks post-immunization than did MVA-*gag* (MVAΔ*udg-gag*: range = 0.12–0.46, median = 0.21; MVA-*gag*: range = 0–0.18, median = 0.087) (Figure 9D). Effective boosting of both Gag-specific CD8 and CD4 T cell responses was observed at one week following the second immunization (1^st^ booster immunization) in both groups of immunized macaques, but was significantly more pronounced (group median responses were approximately 2-fold higher) in those macaques that were immunized with the MVAΔ*udg-gag* vector as compared to MVA-*gag* (Figure 9A, B, D). No effective boosting was subsequently observed in either group following the third immunization (2^nd^ booster immunization) and likely reflects efficient neutralization of the viral inocula due to the high levels of MVA-specific neutralizing antibodies that were present in all animals this time (see below). Taken together, these data indicate that macaques immunized with a Δ*udg* vaccine vector mounted significantly, albeit modestly, higher frequenicies of transgene-specific CD8 and CD4 T cell responses at various times following both primary and booster immunizations, as compared to those animals immunized with a control *udg* ^+^ vector.

### Immunization of rhesus macaques with MVAΔ*udg-gag* and MVA-*gag* elicits similar titers of HIV Gag-specific antibodies

**Figure 10:**
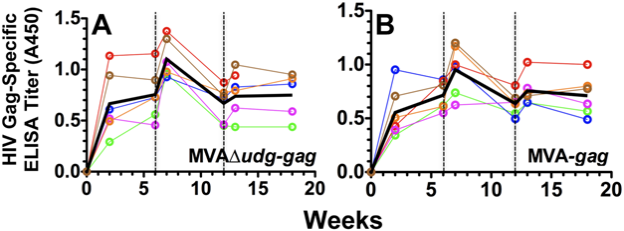
Immunization of rhesus macaques with MVAΔ*udg-gag* elicits HIV Gag-specific antibody responses that are of magnitudes similar to those elicited by MVA-*gag*. Rhesus macaques (N = 6/group) were immunized at 0, 6, and 12 weeks with MVAΔ*udg-gag* or MVA-*gag* (2×10^8^ PFU per immunization). At the indicated times, plasma samples were assayed to determine the titers of HIV Gag-specific binding antibodies via ELISA utilizing recombinant HIV Gag protein as the coating antigen, as described. Symbols represent the mean (of duplicate) absorbance (A450nm) values that were determined from 1∶25 dilutions of plasma samples from individual macaques; black lines denote group mean absorbance values. Immunizations are denoted by vertical dashed lines. Statistical comparison of groups immunized with MVAΔ*udg-gag* vs MVA-*gag* was performed at each timepoint via non-parametric Mann-Whitney analysis and did not result in any significant (P≤0.05) differences.

Plasma samples from macaques immunized with MVAΔ*udg-gag* or MVA-*gag* were assayed to determine whether the relatively higher frequencies of Gag-specific CD4 T-helper cellular responses that were elicited by MVAΔ*udg-gag* correlated with higher levels of Gag-specific antibodies *in vivo*. Titers of HIV Gag-specific binding antibodies were determined by ELISA utilizing recombinant baculovirus-expressed HIV Gag protein as the coating antigen and are shown for macaques immunized with MVAΔ*udg-gag* (Figure 10A) and MVA-*gag* (Figure 10B). Levels of Gag-specific antibodies were similarly detected in both immunization groups beginning two weeks following primary immunization. Effective boosting of these titers was observed in all macaques following the second immunization (1^st^ booster immunization), but to a lesser extent following the third immunization (2^nd^ booster immunization). Comparison of Gag-specific ELISA titers between immunization groups at any given time following immunization with MVAΔ*udg-gag* versus MVA-*gag* revealed no significant differences (Mann-Whitney).

### Immunization of rhesus macaques with MVAΔ*udg-gag* and MVA-*gag* elicits similar titers of vector-specific antibody responses

**Figure 11:**
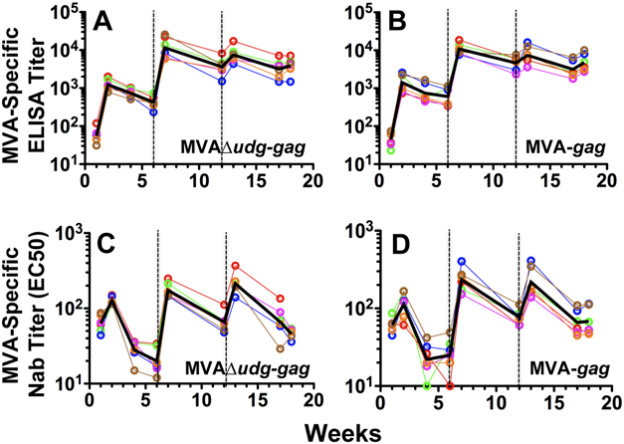
Immunization of rhesus macaques with MVAΔ*udg-gag* elicits MVA-specific antibody responses that are of magnitudes similar to those elicited by MVA-*gag*. Rhesus macaques (N = 6/group) were immunized at 0, 6, and 12 weeks with MVAΔ*udg-gag* or MVA-*gag* (2×10^8^ PFU per immunization). At the indicated times, heat-inactivated plasma samples were assayed to determine the titers of MVA-specific binding antibodies (A, B) via ELISA utilizing whole MVA virions as the coating antigen, or MVA-specific neutralizing antibodies (C, D) utilizing the MVA-*lacZ* neutralization assay, as described. Symbols represent data from individual macaques; black lines denote group geometric mean titers. Immunizations are denoted by vertical dashed lines. Statistical comparison of groups immunized with MVAΔ*udg-gag* vs MVA-*gag* was performed at each timepoint via non-parametric Mann-Whitney analysis and did not result in any significant (P≤0.05) differences.

Plasma samples from macaques immunized with MVAΔ*udg-gag* or MVA-*gag* were assayed to determine whether the abrogation of late viral gene expression during *in vivo* infection, conferred via deletion of *udg* from MVAΔ*udg-gag*, might attenuate the magnitude of antibody responses directed against the MVA vector itself. Endpoint titers of MVA-binding antibodies were determined by ELISA utilizing whole MVA virions as the coating antigen, and are shown for macaques immunized with MVAΔ*udg-gag* (Figure 11A) and MVA-*gag* (Figure 11B). MVA-specific neutralizing antibody (NAb) titers (EC50) were determined by utilizing a plate-based MVA-*lacZ* infection-inhibition assay (which has relatively higher sample throughput as compared to the flow cytometry-based MVA-*gfpzeo* assay used above in our murine studies) and are shown for macaques immunized with MVAΔ*udg-gag* (Figure 11C) and MVA-*gag* (Figure 11D). Within each immunization group, MVA-specific antibody titers (both binding and neutralizing) were detected following primary immunization and exhibited significant boosting (≥1-log) following the second immunization (1^st^ booster immunization) and diminished boosting following the third immunization (2^nd^ booster immunization). Comparison of MVA-specific ELISA titers, or MVA-specific NAb titers, between groups of macaques at any given time following immunization with MVAΔ*udg-gag* versus MVA-*gag* revealed no significant differences in the antibody responses that were elicited by the *udg*-deletion and *udg* ^+^ vectors.

## Discussion

There is a substantial need to improve the immunogenicity of MVA-based vaccines, which are currently being developed for use against a number of prominent infectious diseases including AIDS, malaria, and tuberculosis, as well as human cancers. Engineered MVA vectors, which help focus cellular immune responses away from vector-specific poxvirus antigens and towards heterologous antigens-of-interest, or which engender weaker vector-specific neutralizing antibody responses, should exhibit greater utility than vectors derived from parental MVA for use as vaccines. In the present study, we describe a genetic system that allows for the generation and propagation of MVA mutants that have deletions for essential viral genes, as demonstrated for the deletion of the poxvirus uracil-DNA-glycosylase gene from the MVA genome. We provide a proof-of-concept that MVAΔ*udg* elicits CD8+ T cell responses against a restricted repertoire of vector antigens *in vivo*, as compared to wild type MVA. Furthermore, we demonstrate in a relevant non-human primate model that a *udg* **^−^** AIDS vaccine vector, MVAΔ*udg-gag*, elicited 2–4-fold higher frequencies of HIV Gag-specific CD8 and CD4 T cells in immunized macaques, as compared to a *udg* ^+^ control vector (MVA-*gag*) that expresses an identical HIV *gag* transgene.

The genetic system we describe was predicated upon our identification of the DF-1 chicken embryo fibroblast cell line as being fully permissive for MVA infection. The DF-1 cell line possesses a number of desirable attributes that make it a convenient cellular substrate for the production of MVA recombinants in the research laboratory. These include: MVA growth comparable to that achieved with primary CEF cultures, the ability to plaque MVA on DF-1 cells, and the ability to generate DF-1-derived cell lines that stably express poxvirus genes and complement the growth of essential-gene deletion MVA mutants. In addition, the DF-1 cell line possesses numerous attributes that make it an attractive alternative to primary CEF cultures for the production of clinical-grade lots of MVA-based vaccines. The DF-1 cell line is a spontaneously-immortalized cell line derived from CEFs prepared from line 0 chicken eggs [40]. These cells lack endogenous avian retroviral sequences and exhibit a non-transformed phenotype [40]. Indeed, based on their lack of spontaneous transformation, DF-1 cells are used to score for transformation by potential oncogenes in avian tumorigenicity models [41]. Regulatory approval of the DF-1 cell line, or derivatives thereof, as a cell substrate for use in cGMP manufacture of MVA-based vaccines would obviate the existing need for recurrent certification of primary CEF cultures, which exhibit finite lifespans in culture, and might enable new approaches to optimize the yield and cost-effectiveness of such vaccines.

Replication-defective versions of other viral vectors [35], [37], [38], [56] have been developed for use as vaccine vectors, in large part to increase vector safety. In contrast, MVA has historically exhibited a favorable safety profile as a smallpox vaccine [57] and is unlikely to overcome its host-range restriction during human infection due to the multiple gene deletions it acquired during its derivation [3]. Even so, targeted deletion of an essential gene(s) from MVA will only increase the safety of such MVA vectors by further reducing any potential for complementation *in vivo*.

Interestingly, an approach to generate replication-defective vectors from MVA has not been pursued. This is likely because parental MVA undergoes an abortive growth cycle in most cell types [4], [5], [6], [7] and is commonly propagated on primary CEFs, which, because of their finite lifespan in culture, are not suitable for stable genetic complementation of mutant viruses. Despite reports that the continuous BHK-21 cell line is fully permissive for MVA replication [6], [7], this cell line has not, to our knowledge, been utilized for genetic complementation of MVA mutants.

Thus, having identified a new cell line that is amenable to genetic complementation strategies for MVA, we proceeded to generate a DF-1-derived cell line that constitutively expresses *udg* ^MVA^ and to use this cell line to complement deletion of *udg* from the MVA genome. Our targeting of *udg*, rather than another essential MVA gene, was based partly on initial studies by Holzer and co-workers that demonstrated the feasibility of trans-complementation of a *udg*-deletion in vaccinia virus, and that a *udg*-deletion mutant of vaccinia virus, which also expressed the prM and E structural proteins of tick-borne encephalitis virus (TBE), was better able to protect mice against lethal TBE challenge than was a replication-competent VV recombinant [39], [52] [58]. While the immune mechanisms that mediated this enhanced protection, which potentially include increased levels and/or duration of antigen synthesis from early viral promoters, greater efficiency of antigen processing and/or presentation within infected cells, or augmented cross-presentation of antigens due to the induction of apoptosis in infected cells [30], [59], [60], [61], [62], were not determined, their *in vivo* observations encouraged our studies to delete *udg* from MVA, toward the goal of generating an improved MVA vaccine vector.

Deletion of *udg* from MVA resulted in a virus (MVAΔ*udg*) that is genetically blocked prior to viral DNA synthesis and late gene expression during infection of non-complementing DF-1 fibroblasts. In this regard, the DNA-negative and late gene-negative phenotypes exhibited by MVAΔ*udg* during infection of DF-1 cells are analogous to those observed during infection of non-complementing cells with a VVΔ*udg* mutant [52], [54]. Thus, the growth of *udg* **^−^** MVA was restricted to the trans-(*udg*)-complementing DF-1-derived cell line. As such, restoration of *udg* to a *udg* **^−^** MVA virus, via genetic recombination, and selection for virus growth on non-complementing cells should provide a powerful system to generate and isolate recombinant MVA viruses, as has been described analogously for the rescue of *udg* **^−^** vaccinia recombinants [58].

With regard to the generation of MVA-specific cellular immune responses, we hypothesized that genetic preclusion of late MVA gene expression, via *udg* deletion, would effect a focusing of T cell responses towards antigens expressed early, rather than late, during the MVA infection cycle. Our characterization of the CD8+ T cell responses elicited in mice following immunization with either MVA or MVAΔ*udg* viruses demonstrates, as proof-of-concept, that genetic abrogation of MVA late gene expression results directly in the generation of a restricted repertoire of vector-specific CD8+ T cells that is biased in favor of antigens that are expressed with early, rather than late, kinetics. This was evidenced by the reductions in CD8 T cell responses against determinants encoded within the viral A3L antigen, a known late gene product, as well as the viral A19L antigen, following immunization with MVAΔ*udg*.

With regard to the generation of A3L_270–277_–specific CD8 T cells, there are potentially two sources of the amino acid determinant present during immunization – one from input virion core proteins (in both infectious and non-infectious virus particles) and the second from A3L that is synthesized *de novo* in MVA-, but not MVAΔ*udg*-infected cells. As such, the capacity to prime restricted CD8+ T cells by MVA or MVAΔ*udg* may be expected to vary as a function of the input immunization dose. Indeed, our observations bear this out, as A3L_270–277_–restricted CD8+ T cells were elicited by MVA following both low (10^6^ PFU) and high (10^8^ PFU) dose immunizations, but were only elicited by MVAΔ*udg* following the high dose immunization. In contrast, A19L_47–55_-restricted CD8+ T cells were not generated following immunization with MVAΔ*udg* at either high or low doses of input virus. While relatively little is currently known about this gene, our model predicts that A19L is a late gene that encodes either a non-structural protein, or a structural protein that does not contain amino acid residues 47–55, such that the A19L_47–55_ determinant is not present in the viral inoculum. Taken together, these findings indicate that MVAΔ*udg* elicits a restricted repertoire of vector-specific CD8+ T cell responses, but suggest that the effectiveness of such a genetic approach to focus CD8+ responses away from late viral gene products may be limited under conditions of high-dose immunization due to the potential to cross-prime CD8+ T cells with input viral antigens.

However, compensatory increases in the frequencies of CD8+ T cell responses directed against either of two early viral determinants (B8R_20–27_, K3L_6–15_) were not observed following immunization of mice with MVAΔ*udg*. In part, this may reflect a model-specific effect in that the early B8R_20–27_ determinant already constitutes the immunodominant CD8+ T cell determinant of MVA in C57Bl/6 mice and, as such, may not be amenable to further enhancement via ‘repertoire focusing’. In an analogous immunization model utilizing MHC-selected (*Mamu* A*01^+^) rhesus macaques, we have similarly observed that deletion of *udg* did not augment the frequencies of CD8 T cells that were elicited by either of two known immunodominant SIV determinants expressed from the MVA vector [63]. Alternatively, genetic abrogation of viral late gene expression may simply reduce the number of different vector-specific determinants that elicit CD8+ T cell responses, rather than the magnitudes of the residual responses.

To address this question directly in a relevant vaccine setting, we conducted an immunization trial in MHC-diverse rhesus macaques in which the levels of transgene-specific T cells that were elicited by Δ*udg* and *udg* ^+^ MVA vectors that express an identical HIV *gag* transgene could be compared directly. As shown, immunization of macaques with MVAΔ*udg-gag* resulted in the generation of 2–4-fold higher frequencies of HIV Gag-specific CD8 and CD4 T cells, as compared to MVA-*gag*, at the times of peak T cell responses following both primary and booster immunizations. These data demonstrate that, as a vector for immunizing against a heterologous antigen, the *udg* **^−^** recombinant was modestly more immunogenic than the corresponding *udg* ^+^ control.

With respect to the generation of transgene-specific humoral immune responses, we reasoned that the higher frequencies of Gag-specific CD4 T-helper cellular responses that were elicited in macaques by MVAΔ*udg-gag* might contribute to the generation of relatively higher titers of Gag-specific antibodies. However, our results showing the generation of Gag-specific antibodies with similar titers and with similar kinetics, following immunization of macaques with MVAΔ*udg-gag* or MVA-*gag* recombinants, indicate that deletion of *udg* had neither beneficial nor detrimental effects in this regard. Thus, the positive effects of *udg* deletion on transgene-specific immunity were observed for T cell, rather than humoral, immune responses.

With regard to the generation of MVA-specific humoral immune responses, we originally hypothesized that abrogation of late gene expression, via *udg* deletion from MVA vectors, would result in the generation of reduced levels of vector-specific neutralizing antibodies. Analogous to the dual sources of CD8+ T cell determinants for viral structural antigens, there are potentially two sources of relevant B cell epitopes that can elicit vector-specific neutralizing antibody responses. These include virion structural proteins that are either associated with input virus or are synthesized *de novo* within infected cells. Because such structural proteins are typically synthesized during infection with predominantly late kinetics, it was reasonable to originally hypothesize that MVAΔ*udg* might elicit reduced titers of vector-specific neutralizing antibodies, as compared to wild type MVA.

However, our results indicate that deletion of *udg* had no demonstrable effect on the overall levels of MVA-specific antibodies that were generated *in vivo*, in either mice or rhesus macaques, following immunization with Δ*udg* or *udg* **^+^** vectors. In mice, no significant differences were observed between the titers of MVA-specific NAbs that were elicited by either MVAΔ*udg* or MVA following immunization over a 4-log range of input virus (10^4^–10^8^ PFU). This relatively shallow dose-response relationship between input virus dose and ensuing vector-specific NAb titers suggests that these NAb responses are fully generated by relatively low amounts (containing ≤10^4^ PFU) of input viral inocula. Whether a similar dose-response relationship would be observed following immunization of mice by different anatomical routes, or in other species, is under investigation. Additionally, the magnitudes and kinetics of MVA-specific antibody responses (both virion-binding and neutralizing) were virtually identical between Δ*udg* and *udg* ^+^ vectors following both primary and booster immunizations of rhesus macaques.

Our observations that Δ*udg* and *udg* ^+^ MVA vectors elicit similar levels of vector-specific antibodies *in vivo* are consistent with those of Ober, *et al* that showed comparable levels of vaccinia virion-binding antibodies in mice following single-dose immunization with replication-defective (Δ*udg*) vaccinia virus (dVV-L, strain Lister), replication-competent vaccinia (VV-L), or MVA [64]. Interestingly, a subsequent study by this group showed the levels of vector-specific antibodies elicited in mice by MVA to be relatively lower than those elicited by the replication-defective dVV-L virus [65].

Taken together, our results suggest that any contribution of *de novo* synthesized viral late gene products to the net MVA-specific antibody response *in vivo* is negligible. Given the demonstration that vaccinia-specific antibodies are both necessary and sufficient to confer protection against virulent monkeypox challenge in non-human primates [66], our finding that levels of MVA-specific NAbs may be driven predominantly by input viral antigens should help inform our understanding of the mechanism by which next-generation smallpox vaccines elicit protection.

In conclusion, we feel that the genetic system that we have developed provides a platform for the development of a new generation of MVA-based vaccine vectors that may be safer and more efficacious than those currently available. The growth of such vaccine vectors on a continuous, consistent, expandable, and more-readily-certifiable cell line should ultimately facilitate their production for use in humans. The establishment of a genetic complementation system for MVA now allows for genes, other than *udg*, to be deleted from the viral genome to pursue hypothesis-driven efforts to maximize vector immunogenicity. As a result, the methods we have developed increase the facility with which the biology of MVA and its interactions with host cells and host organisms can now be studied. The knowledge gained from such studies promises to enhance our ability to derive novel MVA variants with enhanced immunogenic properties for use as safe and effective vaccines.

## Materials and Methods

### Ethics Statement

All animal studies were performed in accordance with animal welfare protocols approved by Emory University.

### Viruses

MVA (p579), generously provided by B. Moss (National Institutes of Health), was amplified on primary CEFs or DF-1 chicken embryo fibroblasts as indicated. The conditional-lethal vaccinia virus mutant *ts*4149, which harbors a temperature-sensitive mutation in the D4R (uracil-DNA-glycosylase) open reading frame, was kindly provided by G. McFadden (University of Western Ontario) [51]. Virus stocks were prepared as lysates of infected cells that were subsequently clarified via centrifugation (800 *g*). Infectious titers of virus stocks were determined via TCID_50_ assay on primary CEFs (where indicated) or via plaque assay on DF-1 cell monolayers. During the course of this study, several MVA recombinants were generated and the genotypes of these recombinant viruses, with regard to uracil-DNA-glycosylase (*udg*), *gfpzeo* (*gz*), and HIV-*gag* transgene (*gag*) are summarized as follows: vDG001 (*gz* ^+^), vDG013 (Δ*udg*, *gz* ^+^), vDG014 (Δ*udg*, *gz* ^+^); vDG027 (Δ*udg*, *gz* ^−^); vDG021 (Δ*udg*, *gz* ^+^, *gag* ^+^); vDG022 (*udg* ^+^, *gz* ^+^, *gag* ^+^). Viruses vDG021 and vDG022 express a synthetic, codon-optimized, subtype-B consensus HIV-*gag* gene (GenBank AY531263) and *gfpzeo* from independent early (modified H5) viral promoters recombined into the MVA deletion-III site. Virus MVA-*lacZ* expresses *lacZ* under the control of a modified H5 viral promoter recombined into the MVA deletion-III site. Uracil-DNA-glycosylase-deletion recombinants were propagated and titered on complementing DF-1-derived cell lines (as described). For use in animal studies, virus stocks underwent purification via centrifugation through a 36% sucrose cushion prior to resuspension in PBS and titration via plaque assay on a commonly permissive cell line.

### Cells

The UMNSAH/DF-1 (DF-1) chicken embryo fibroblast cell line [40], [41], kindly provided by H. Varmus (Memorial Sloan-Kettering Cancer Center, New York, NY) and currently available through ATCC (#CRL-12203; Manassas, VA), was propagated in Dulbecco's Modified Eagle's Medium (DMEM) that was supplemented with 10% heat-inactivated fetal bovine serum (FBS; HyClone, Logan, UT), 100 I.U./ml penicillin (PEN), 100 µg/ml streptomycin (STREP), and 2 mM L-glutamine (GLUT). Primary chicken embryo fibroblasts (CEF) prepared from 8–11 day embryos were obtained from Charles River SPAFAS, Inc. (Preston, CT) and propagated in Basal Medium Eagle (Gibco/Invitrogen) that was supplemented with 5% FBS, PEN, STREP, and GLUT. All DF-1-derived cell lines (described below) were propagated in DF-1 growth medium that was supplemented with 300 µg/ml G418 Sulfate. BHK-21 cells were obtained from ATCC (#CCL-10) and propagated in Eagle's MEM supplemented with 10% FBS, PEN, STREP, GLUT, non-essential amino acids, and 1 mM sodium pyruvate. HeLa cells were obtained from ATCC and propagated in suspension culture in MEM Spinner media (Quality Biologicals, Inc) supplemented with 5% heat-inactivated horse serum, PEN, STREP, and GLUT. All tissue culture growth media and supplements were obtained from Mediatech (Herndon, VA) unless noted otherwise. Zeocin was purchased from Invitrogen (Carlsbad, CA).

### Generation of DF-1-derived cell lines

To allow generation of DF-1-derived cell lines that constitutively express UDG^MVA^, the pCAN gene-expression vector was constructed for use in avian cells by subcloning a 1.7 kb CMV IE-chicken ß-Actin promoter/enhancer element (kindly provided by J. Jacob, Emory Vaccine Center [67]) into pNEB193 (New England Biolabs, Beverly, MA) to yield pCMVACT193. Subsequently, a 2.3 kb BamHI SV40-Neo^R^ expression cassette was subcloned from pIRES (BD Biosciences Clontech, Palo Alto, CA) into pCMVACT193 to generate pCAN (CMV IE-chicken ß-Actin/Neo^R^).

The *udg* ORF (MVA nucleotides 92,417–93,073; Genbank accession U94848) was amplified via polymerase chain reaction from genomic MVA DNA with forward primer 5′-tctcgagctcaATGAATTCAGTGACTGTATCA-3′ (initiator methionine codon underlined) and reverse primer = 5′-cgcggtaccgtcTTAATAAATAAACCCTTGAGC-3′ (translation termination underlined; *udg* ORF in capital letters) and cloned into pCR2.1 (Invitrogen) to yield p2.1*udg*ORF. The *udg* ORF was subsequently re-amplified via PCR with forward primer 5′-aaagcttagatct**gccacc** ATGAATTCAGTGACTGTA-3′ (partial Kozak consensus in bold, initiator methionine codon underlined) and reverse primer 5′-agcggccgctacgtaTTAATAAATAAACCCTTG-3′ (translation termination underlined) to incorporate a partial translation initiation consensus sequence immediately preceding the *udg* ORF [68]. This PCR product was cloned into the pCR-Blunt II-TOPO vector (Invitrogen) to yield pDG100 and its nucleotide identity was confirmed via DNA sequencing. The *udg* ORF was subsequently positioned under the control of the CMV IE-chicken ß-Actin promoter/enhancer element in the pCAN expression vector to yield pCAN*udg*.

DF-1-derived cell lines that constitutively express UDG^MVA^ were generated by calcium phosphate-mediated transfection of the *udg*-expression plasmid pCAN*udg* into DF-1 cells followed by clonal selection of G418^R^ cells. G418^R^ cell lines were screened for their ability to complement the growth of *ts*4149, a vaccinia virus mutant that harbors a temperature-sensitive mutation in the *udg* (D4R) gene, at the non-permissive temperature of 39.5°C [51], [53]. The G418^R^ cell line that exhibited the highest level of complementation, designated CAN20, was subsequently used to generate and propagate *udg*-deletion recombinants of MVA (see below).

### Construction of MVA transfer vectors

#### MVA deletion site III vectors: pG06dH5, pG06dH5*gz*

A vector to direct recombination of two gene expression cassettes under the control of early viral promoters into MVA deletion site III was constructed by replacing the synthetic double promoter in plasmid pG06 [69] with bidirectional early (modified H5) viral promoters. Two early viral (modified H5) promoters flanked by restriction endonuclease sites (PmeI, ApaI, PacI, Eco47III←pH5– StuI; StuI –pH5→NaeI, MluI, SacII, SacI) were generated by pairwise ligation of oligonucleotides H5For2+H5Rev2, H5Rev2+H5Rev3: H5For2 = 5′-agtttaaacaagggcccaactcgagaattaattaaaaagcgctTATTTAT*GATTATTTCTCGCTTTCAATTTAACACAA*-3′, H5Rev2 = 5′-aggcctAAAAATTGAAAATAAATACAAAGGTTCTTGAGGG*TTGTGTTAAATTGAAAGCGAGAAATAATCATAAATAA*-3′, H5Rev3 = 5′-agagctcaatcgcgaaaccgcggaaacgcgtAAGCCGGCTATTTATGA*TTATTTCTCGCTTTCAATTTAACACAA*-3′ (upper case denotes promoter sequence, italics denote regions of oligonucleotide complementarity). Single strand overhangs of ligated oligonucleotides were filled in using Taq polymerase and these dsDNA products were cloned into pCR2.1 (Invitrogen) to generate vectors pTA-H5anti and pTA-H5sense, respectively. Modified H5 promoters were assembled into a bidirectional promoter element via ligation of the 3.1 kB StuI/BglII fragment of pTA-H5sense and 1.0 kB StuI/BglII fragment of pTA-H5anti to generate pTA-dH5. The double synthetic promoters of pG06 were removed via digestion with PmeI and SacI and replaced with an EcoRV-SacI fragment containing the bidirectional modified H5 promoter element from pTA-dH5 to yield MVAΔIII transfer vector pG06dH5.

The *gfpzeo* ORF was amplified from pTracer-SV40 (Invitrogen) via PCR with forward primer 5′-aatcgcgaATGGCGGTAGAAAAAATG-3′ and reverse primer 5′-tacgt*agaaaaa*TCAGTCCTGCTCCTC-3′ (upper case denotes *gfpzeo* ORF, italics denote early vaccinia virus early gene transcriptional stop signal [70]). The 1.1 kB NruI/SnaBI-digested *gfpzeo* fragment was cloned into NruI/SnaBI-digested pG06dH5 to position the *gfpzeo* ORF under the control of –pH5→to yield vector pG06dH5*gz*. The transfer vector pG06dH5*gz* was subsequently used to generate the viral recombinant MVA-*gz* (vDG001) as described below.

#### *udg* deletion vector: pΔ*udg*dloxPH5*gz*

Genomic regions flanking the *udg* ORF were amplified via PCR from MVA DNA. The left flanking region was amplified with forward primer 5′-aagagctcATATTGTGACTCCAGATACATATGGA-3′ and reverse primer 5′-aaactagtCATTATATCAAATTAGATACCTTTTTATACG-3′; the right flanking region was amplified with forward primer 5′-aactcgagAATGCTTTAGTGAAATTTTAACTTGTGTTC-3′ and reverse primer 5′-aaggtaccTCCTAGTACCTACAACCCGAAGAG-3′ (upper case denotes MVA sequences, lower case denotes appended restriction endonuclease sites). These genomic regions flanking *udg* were individually cloned into the pCR-BluntII-TOPO vector and verified via DNA sequencing. The right and left *udg* flanks were sequentially cloned into pBluescriptII(SK+) (Stratagene) as XhoI-KpnI and SacI-SpeI fragments, respectively, to yield plasmid pBS*udg*LFRF. To position a *gfpzeo* expression cassette between two loxP sites, plasmid pMS102 (kindly provided by J. Murray, University of Cambridge [71]) was first digested with SnaBI and EcorRV and religated to render a unique XbaI site between the two loxP elements in this plasmid construct (pMSΔ102). A 1.2 kb StuI-SnaBI pH5-*gfpzeo* expression cassette from pG06dH5*gz* was blunt cloned in both leftward and rightward orientations into the Klenow-filled XbaI site in pMSΔ102 to yield plasmids pMSΔ102H5GZ.1, -.4, respectively. To construct transfer vectors that direct the replacement of the *udg* ORF with a *gfpzeo* expression cassette, SpeI-BglII fragments (containing loxP-flanked pH5-*gfpzeo*) were subcloned from pMS102ΔH5GZ.4 and pMSΔ102H5GZ.1 into SpeI-BamHI linearized pBS*udg*LFRF to generate plasmids pΔ*udg*dloxPH5GZ-A, -B, respectively. Transfer vectors pΔ*udg*dloxPH5GZ-A and pΔ*udg*dloxPH5GZ-B, which differ only in the orientation of the *gfpzeo* expression cassette, were subsequently used to generate MVAΔ*udg* recombinants vDG013 and vDG014, respectively, as described below.

### Generation and isolation of recombinant MVAs

Recombinant MVAs were generated via homologous recombination by infecting 2×10^6^ permissive cells at MOI = 0.05 for 1.5 hours followed by transfection of 1 µg MVA transfer vector (supercoiled plasmid DNA) via Effectene (Qiagen, Valencia, CA) according to the manufacturer's protocol. At 48 hours following infection, progeny viruses were released from infected cells via lysis (1 freeze/thaw cycle followed by sonication) and were plated at various dilutions onto monolayers of permissive cells (DF-1 or DF-1-derived cell lines, as indicated). Recombinant *gfpzeo* ^+^ viruses were selected for by application of an agarose overlay (1% low-melting agarose/1XDMEM (GibcoBRL)) that was supplemented with 2% FBS, 100 I.U./ml PEN, 100 µg/ml STREP, and 200 µg/ml Zeocin (Invitrogen). Recombinant viruses were identified as foci of GFP^+^ cells that were readily detected by 2 days following infection using fluorescence microscopy. Recombinant viruses were plaque purified through at least 3 rounds of Zeocin selection and analyzed by diagnostic Southern blots to ensure clonality. MVA recombinant MVA-*gz* (vDG001) was generated and propagated on DF-1 fibroblasts. Udg-deletion recombinants vDG013 and vDG014 were generated and propagated on the *udg*-complementing CAN20 cell line.

### Southern blotting

DNA for Southern blot analyses was isolated from infected cells following cell lysis in buffer containing 20mMTrisHCl (pH = 8.0), 10 mM EDTA, and 0.75% SDS. Cell lysates were incubated overnight with 20 mg/ml proteinase K at 37°C and DNA was isolated by phenol/chloroform extraction and ethanol precipitation. Following restriction endonuclease digestion, DNA samples were electrophoresed through 0.7% agarose gels, and transferred to Nytran SuPerCharge membrane (Schleicher and Schuell) according to standard procedures [72]. Plasmid probes were labeled with digoxygenin-dUTP via random-prime labeling (Roche; Indianapolis, IN), hybridized to membranes [73] and immunologically detected via chemiluminescence with CSPD substrate (Roche) and BioMax film (Kodak).

### Determination of viral growth kinetics

Primary CEFs (p11), DF-1 fibroblasts, and BHK-21 cells were infected with 3 infectious units of MVA per cell and incubated at 37°C in low serum (2% FBS) growth media. Infected cell cultures were frozen at indicated times following infection and subsequently thawed, sonicated, and clarified by centrifugation (800 *g*). MVA titers were determined via TCID_50_ assay on CEFs (p13) or plaque assay on DF-1 fibroblasts, as indicated.

### Metabolic labeling of infected-cell proteins

Two million cells were infected with MVA or MVAΔ*udg* (vDG014) at a ratio of 10 PFU per cell. Following 90 min adsorption, cells were washed with PBS and incubated in low-serum (2%) DMEM culture medium in the absence or presence of 150 µM Cytosine ß-D-Arabino-Furanoside (AraC; Sigma), as indicated. For metabolic labeling, cells were washed once with PBS and then incubated for 30 min (prior to indicated timepoint) at 37°C with 1 ml Methionine-free DMEM (Sigma) that was supplemented with 2% FBS, 100 I.U./ml penicillin, 100 µg/ml streptomycin, 2 mM L-glutamine, 10 mM HEPES (pH 7.4), and 90 µCi ^35^S-TransLabel (ICN) per 2×10^6^ cells. Proteins were resolved by SDS-PAGE and labeled proteins were visualized via autoradiography.

### BrdU-labeling of DNA

DF-1 cells were grown on glass coverslips and infected with MVA or vDG020, a *udg*-deletion MVA recombinant that expresses RFP and puromycin-resistance gene products from early modified H5 promoters at the MVAΔIII locus) at a ratio of 10 PFU per cell, or were mock-infected, in low-serum (2% FBS) culture medium in the presence or absence of 150 µM AraC. Cells were labeled between 2–6 hours following infection via incubation with culture medium that was supplemented with 10 µM BrdU (Becton-Dickinson, BD). Cells were then fixed and permeabilized (cytofix/cytoperm, BD), treated with DNase (300 µg/ml in DPBS with Ca^+2^, Mg^+2^ for 1 hour at 37°C), stained with primary anti-BrdU-FITC or isotype (mouse IgG1-FITC) control antibodies (BD) and secondary anti-mouse-Ig-fluorescein (Amersham) and observed via fluorescence microscopy.

### Murine Studies

#### Intracellular Cytokine Assay of MVA-Specific CD8+ T Cells

Female C57Bl/6J mice (The Jackson Laboratory, Bar Harbor, ME) aged 6–8 weeks were immunized by intraperitoneal injection of 10^6^ PFU MVA or MVAΔ*udg* (vDG027), as indicated. Seven days later, the reactivity of CD8+ splenocytes to a panel of individual peptides representing previously identified vaccinia virus CD8+ T cell epitopes [55], was determined by intracellular cytokine staining and flow cytometric analysis. Briefly, dissected spleens were manually disrupted into cellular suspensions and red blood cells were lysed using NH_4_Cl buffer (Sigma). Remaining cells were enumerated and samples of one million splenocytes were stimulated with 0.5 µM peptide in the presence of GolgiPlug (Becton Dickinson [BD]) for 5 hours at 37°C. Peptides used for stimulation represent H2^b^-restricted vaccinia virus CD8+ T cell determinants A3L_270–277_ (KSYNYMLL), A42R_88–96_ (YAPVSPIVI), B8R_20–27_ (TSYKFESV), K3L_6–15_ (YSLPNAGDVI), or A19L_47–55_ (VSLDYINTM) [55], [74]. Following stimulation, cells were surface-stained with fluorescently labeled antibodies FITC-CD3 (2C11) and PerCP-CD8 (53-6.7), then permeabilized using cytofix/cytoperm (BD) and stained with antibody APC-IFNγ (XMG1.2); all antibodies were purchased from BD. Finally, cells were fixed with 2% formaldehyde, 0.1% sodium azide in PBS, and analyzed on a FACScalibur (BD). For analysis (FlowJo, Treestar, Inc), splenocytes were gated on lymphocytes by forward/side scatter and T cells by CD3 positivity prior to gating on CD8 and IFNγ.

#### Determination of MVA-Specific Neutralizing Antibody Titers

C57Bl/6J mice (The Jackson Laboratory, Bar Harbor, ME) aged 6–8 weeks were immunized by intraperitoneal injection with MVA or MVAΔ*udg* (vDG027) at a dose of 10^4^–10^8^ PFU, as specified. At 28 days following immunization, mice were sacrificed and blood collected via cardiac puncture. To prepare serum, blood was allowed to clot overnight at 4°C, clarified by centrifugation, and heat-inactivated at 56°C for 30 minutes. Serum samples were assayed in duplicate by a flow cytometry (GFP)-based neutralization assay adapted from that described by Earl, et al [75]. Briefly, serial 2-fold dilutions of sera were incubated for 1 h at 37°C with a fixed volume of MVA-*gz*, which had been previously determined via titration to infect 25–30% of 1×10^5^ HeLa cells in suspension. Serum-virus mixtures were then added to 1×10^5^ HeLa cells and incubated for 16 h at 37°C. Samples were fixed by adding an equal volume of 1% formaldehyde in PBS, processed on a FACScalibur flow cytometer (BD) and analyzed (FlowJo) to determine the percentages of GFP+ HeLa cells. EC50 values were calculated by nonlinear regression analysis of the replicate dilution series for each sample using Prism 4 software (GraphPad Software, Inc.).

### Rhesus Macaque Studies

#### Immunizations

Male rhesus macaques, aged 3.5–5 years were immunized with a total of 2×10^8^ PFU of MVAΔ*udg-gag* or MVA-*gag* that was delivered by a split route: 1×10^8^ PFU intradermally and 1×10^8^ PFU intramuscularly into alternate thighs.

#### Intracellular Cytokine Assay

Cryopreserved PBMCs were thawed and rested overnight at 37°C in complete cell culture medium. Replicate cultures of 2×10^6^ PBMCs were cultured for 6 hours in the absence (unstimulated) or presence (stimulated) of a single pool of HIV Gag peptides (15-mers, overlapping by 11; NIH AIDS Research & Reference Reagent Program), which were identically matched to the Gag antigen expressed by both MVA vectors, at a final concentration of 2 µg/peptide/ml. Cytokine secretion was blocked during the final 4 hours by the addition of GolgiPlug (BD Biosciences). Cells were stained with Alexa-430 (Invitrogen) to enable subsequent live/dead discrimination, permeabilized and stained with a cocktail of fluorophore-labeled antibodies, and analyzed on an LSR-II flow cytometer (BD Biosciences). The antibody staining panel consisted of CD4-FITC (L200; BDP), IL2-PE (17H12; BDP), CD8ß-ECD (2ST8.5H7; BC), CD69-PECy5.5 (CH/4; CAL); IFNγ-APC (B27; BDP), CD14-APCCy7 (M5E2; BIO), CD20-APCCy7 (2H7; BIO), and CD3-PacificBlue (SP34-2; BDP) [BDP = BD Pharmingen; BC = Beckman Coulter; CAL = Caltag; BIO = Biolegend]. Data were analyzed using FlowJo analysis software (Tree Star, Inc.). The hierarchical gating strategy used to determine the frequencies of cytokine-positive CD8 and CD4 T cells is illustrated (Figure S1). For each T cell∶cytokine combination analyzed, the means of replicate unstimulated PBMC samples were subtracted from the corresponding mean of replicate Gag-stimulated PBMCs to yield a single value per macaque per timepoint. Statistical comparison of MVAΔ*udg-gag* and MVA-*gag* groups was performed by nonparametric Mann-Whitney analysis using Prism software (GraphPad Software, Inc.).

#### Determination of HIV Gag-Specific Binding Antibody Titers

Maxisorb ELISA plates (Nunc, Rochester, NY) were coated overnight (4°C) with baculovirus-expressed recombinant HIV Gag protein (HIV-1_IIIB_p55Gag; #3276; NIH AIDS Research and Reference Reagent Program) in a 50 µl volume per well at a concentration of 1 µg/ml in 0.1 M sodium bicarbonate buffer (pH 9.5). Plates were washed three times with wash buffer (KPL, Inc; Gaithersburg, MD), and blocked with 10% fetal bovine serum in wash buffer for 1 hour at room temperature. Macaque plasma samples were diluted 1∶25 with blocking solution. 50 µl aliquots of diluted plasma were incubated, in duplicate, in wells of the Gag-coated ELISA plates for 2 hours at room temperature. Plates were washed 5 times with wash buffer (200 µl/well) followed by incubation for 45 minutes at room temperature with 50 µl volume per well of the 2° polyclonal anti-rhesus-HRP antibody (Accurate Chemical and Scientific Corp., Westbury, NY) at 1∶8,000 dilution in blocking solution. Plates were washed seven times with wash buffer and incubated with TMB (3,3,5,5′-tetramethylbenzidine) Microwell Peroxidase substrate system (2-C) (KPL, Inc.) at 50 µl per well, for 20 minutes at room temperature. The reaction was stopped by the addition of 1 M phosphoric acid (25 µl per well). Absorbance (450 nm) values were obtained via plate reader, corrected for blank-well absorbance, and the values for replicate samples were averaged. For each individual macaque, post-immunization data were normalized to the pre-immunization baseline by subtraction of the corresponding pre-immunization Gag-ELISA value.

#### Determination of MVA-Specific Binding Antibody Titers

Maxisorb ELISA plates (Nunc, Rochester, NY) were coated overnight with 5×10^6^ PFU of sucrose-purified MVA per well, washed four times with PBS, and blocked 1.5 hours at 37°C with 4% BSA/17% fetal bovine serum in PBS. Serial 1∶2 dilutions of test serum (in duplicate) were incubated in MVA virion-coated plates for 1.5 hours at 37°C. Plates were washed 5 times with PBS (200 µl/well) followed by incubation for 1.5 hours at 37°C with polyclonal anti-rhesus-HRP antibody (Accurate Chemical and Scientific Corp., Westbury, NY) at 1∶10,000 dilution in blocking solution. Plates were washed five times with PBS and incubated with One-Step TMB (3,3,5,5′-tetramethylbenzidine) solution (Pierce, Rockfore, IL) for 20 minutes at room temperature. The reaction was stopped with 4N H_2_SO_4_. A450nm values were obtained via plate reader and were corrected for blank-well absorbance. Corrected absorbance data were analyzed as a function of serum dilution by non-linear regression analysis using Prism software (GraphPad Software, Inc.). For each individual sample, the MVA-specific ELISA titer is reported as the serum dilution corresponding to an A450nm value of 0.3, as interpolated from the fitted curve (R^2^>0.97).

#### Determination of MVA-Specific Neutralizing Antibody Titers

Titers of MVA-specific neutralizing antibodies were determined with an MVA-*lacZ* infection-inhibition assay adapted from Manischewitz, *et al* [76]. This assay, which is performed in 96-well plates, provided a relatively higher throughput assay than did the MVA-*gfpzeo* assay described above, and was therefore employed for analyzing the samples from the macaque immunization trial. Briefly, serial 1∶2 dilutions of heat-inactivated test sera (50 µl volume) were mixed with equal volumes of DMEM containing 2.5×10^4^ PFU MVA-*lacZ* and incubated for 1 hour at 37°C. At the end of this period, 1×10^5^ DF-1 cells were added per well and incubated for an additional 16 hours at 37°C. Culture supernatant was removed and the cells were washed twice with PBS, followed by lysis via addition of 100 µl Reporter Lysis Buffer (Promega) per well and incubation for 15 minutes at room temperature. Plates were centrifuged for 5 minutes and 50 µl aliquots of lysates were transferred into new 96-well plates. ß-gal activity was assayed by addition of 50 µl ONPG solution (4.3 mM ONPG, 0.2 M NaH_2_PO_4_, 0.1 M Na_2_HPO_4_, 4 mM MgCl_2_, 0.1 M ß-mercaptoethanol) and incubation for 30 minutes at 37°C. The reaction was stopped by adding 150 µl 1 M Na_2_CO_3_ per well. Absorbance readings of samples were made at 420 nm and corrected for blank-well absorbance. Corrected absorbance data were analyzed as a function of serum dilution by non-linear regression analysis using Prism software (GraphPad Software, Inc.). For each individual sample, the MVA-specific NAb titer is reported as the serum dilution corresponding to the best-fit EC50 value.

## Supporting Information

Figure S1 Gating Strategy and Exemplary Intracellular Cytokine Assay Data
(0.25 MB PDF)
