# Supplementary material for: Expanding the Repertoire of Modified Vaccinia Ankara-Based Vaccine Vectors via Genetic Complementation Strategies
Source: PLoS One. 2009 May 6;4(5):e5445. doi: 10.1371/journal.pone.0005445 (PMC2674217; doi:10.1371/journal.pone.0005445)
Supplement: Figure S1 — Gating Strategy and Exemplary Intracellular Cytokine Assay Data (0.25 MB PDF) [file pone.0005445.s001.pdf]

**Figure S1**. Gating Strategy and Exemplary Intracellular Cytokine Assay Data

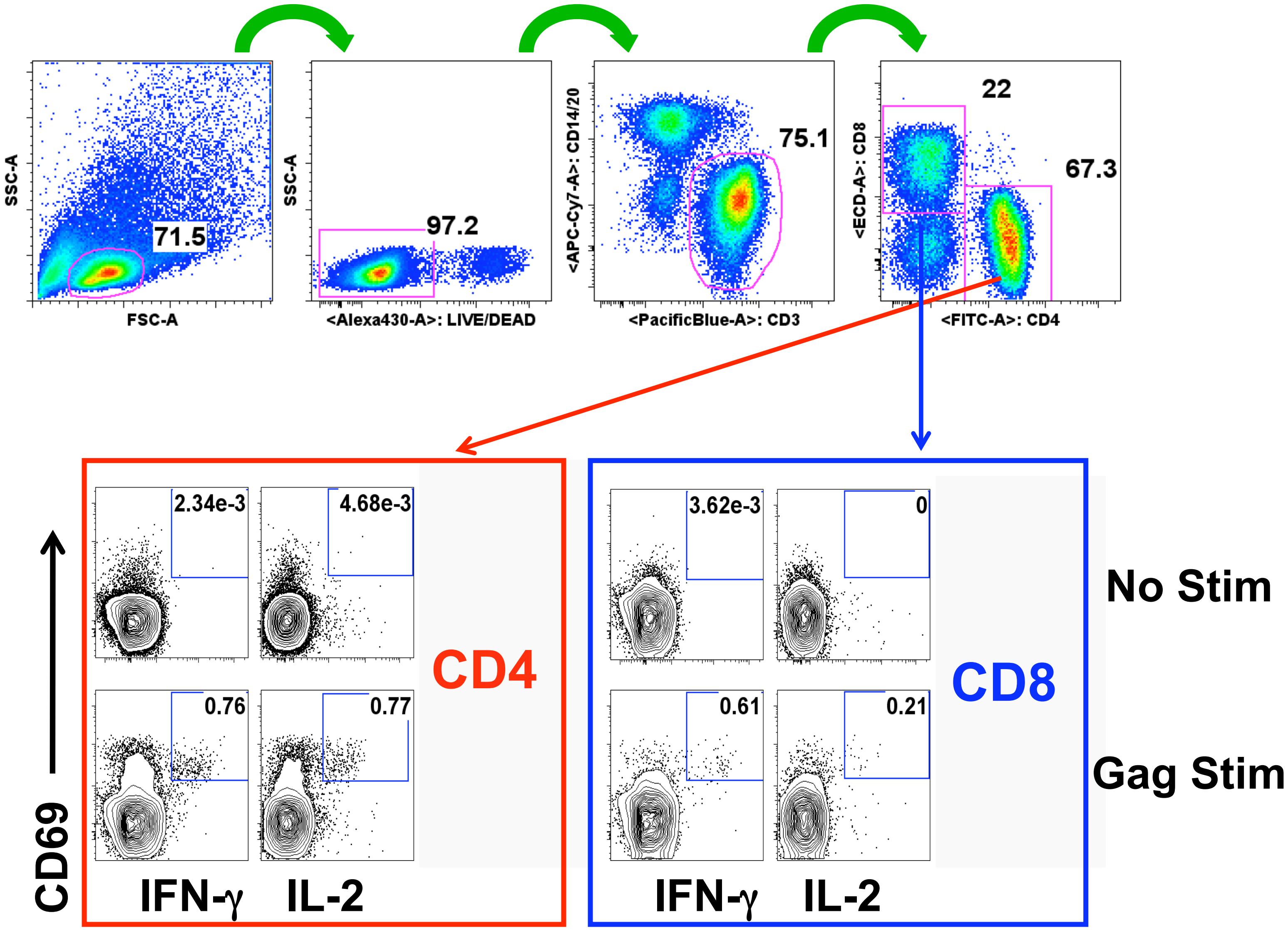

**Figure S1. Gating Strategy and Exemplary Intracellular Cytokine Assay Data**

Macaque PBMC samples were processed for intracellular cytokine assay as described in Materials & Methods. (Top) Representative gating of flow cytometry data is shown: hierarchical gating included a lymphocyte gate based on forward/side light scatter, exclusion of dead (Alexa430-positive) cells, exclusion of monocytes/macrophages (CD14+) and B cells (CD20+) and inclusion of T cells (CD3+), and discrimination of T cells into CD4+ andCD8+ populations. (Bottom) Expression of cytokine (IFN$\gamma$ or IL-2) versus cell activation (CD69+) in PBMCs, from an individual macaque, that had been stimulated with the HIV Gag peptide pool (Gag Stim) or not stimulated (No Stim) . The numbers represent the proportions of CD4 or CD8 T cells that are CD69+Cytokine+.
